# Rituximab for Leucine-Rich Glioma-Inactivated 1 (LGI1) Antibody-Related Super-refractory Status Epilepticus: A Case Report and Literature Review

**DOI:** 10.7759/cureus.81094

**Published:** 2025-03-24

**Authors:** Alawi A Al-Attas, Samar E Eshetaiwe, Mesdah A Alnahdi, Hoda M Nassar, Ahmed H Althobaiti

**Affiliations:** 1 Neurology and Epilepsy Department, King Saud Medical City, Riyadh, SAU; 2 Neurology Department, King Saud Medical City, Riyadh, SAU

**Keywords:** fbds, hyponatremia, immunotherapy, lgi-1 encephalitis, rituximab

## Abstract

Leucine-rich glioma-inactivated 1 (LGI1) encephalitis is a form of autoimmune encephalitis (AE) that presents with memory loss, faciobrachial dystonic seizures (FBDSs), disorientation, psychiatric symptoms, and hyponatremia. Diagnosis is based on clinical presentation, magnetic resonance imaging (MRI), serum or cerebrospinal fluid (CSF) antibody testing, and electroencephalography (EEG) findings. Most available studies on its clinical features and treatment are limited to case reports and series, highlighting the need for a comprehensive understanding and standardized treatment approach. Herein, we report a 61-year-old Saudi retiree with cognitive decline, recurrent right FBDS, generalized tonic-clonic seizures, and persistent hyponatremia who showed a significant improvement after rituximab therapy.

## Introduction

Anti-leucine-rich glioma-inactivated 1 (anti-LGI1) encephalitis is a rare autoimmune encephalitis (AE) characterized by cognitive impairment, psychiatric symptoms, refractory hyponatremia, and seizures, particularly faciobrachial dystonic seizures (FBDSs). It is the fourth syndrome associated with antibodies targeting the voltage-gated potassium channel (VGKC) complex and the second most frequent cause of autoimmune encephalitis, accounting for 11.2% of all cases [1].

LGI1 is essential for regulating neuronal networks by modulating excitability and synaptic transmission, contributing to neuronal network maturation, and interacting with proteins that control synaptic function, whereas mutations or autoantibodies targeting LGI1 are associated with epilepsy, particularly autosomal dominant temporal lobe epilepsy, leading to cognitive impairments and seizures [2]. Anti-LGI1 encephalitis is typically unrelated to malignancies, despite the possibility that it is a paraneoplastic condition. Indeed, the condition primarily affects men in their 60s [3].

FBDSs are the hallmark seizure type, occurring in 40%-71% of cases. While highly specific for LGI1 encephalitis, they are not exclusive to the condition. FBDS involves involuntary dystonic posturing of the arm, followed by facial contraction, lasting 1-30 seconds, typically affecting one arm and the face. They are often underrecognized, occurring up to 200 times per day, with ipsilateral leg involvement in 40% of cases and, occasionally, bilateral presentation [4].

FBDS and subtle seizures typically precede cognitive decline and memory impairment; however, other seizure types, including focal seizures with dyscognitive, autonomic motor, and gelastic features, may also occur. In addition to memory impairment, patients may develop behavioral disturbances such as apathy, disinhibition, impaired social awareness, compulsive behavior, egocentrism, and spatial disorientation. Furthermore, other associated symptoms include insomnia, hyperhidrosis, and hyponatremia [5].

Imaging and laboratory tests may be inconclusive in LGI1 encephalitis because magnetic resonance imaging (MRI) often reveals hippocampal T2 hyperintensity, which is typically bilateral but can be unilateral or even absent in the early disease stages; however, prolonged symptoms are frequently associated with medial temporal changes, and T1 hyperintensities in the basal ganglia have also been reported. Additionally, electroencephalography (EEG) findings may be minimal during FBDS, although longer seizures can exhibit detectable EEG abnormalities, while cerebrospinal fluid (CSF) pleocytosis is present in only 23% of encephalitis cases, and up to 13% of patients with encephalopathy may have normal MRI and CSF findings, despite clear clinical evidence of encephalitis [4,5].

Seizures in LGI1 encephalitis are often resistant to standard anti-seizure medications (ASMs); however, immunotherapy plays a crucial role in the treatment [5]. First-line therapy consists of high-dose corticosteroids, either alone or in combination with plasma exchange (PE) or intravenous immunoglobulin (IVIG). However, improvement is typically observed within two weeks, although full recovery may take up to one year [6].

If first-line therapy is insufficient, second-line treatment includes cyclophosphamide or rituximab, with immunotherapy being more effective than anti-seizure medications (ASMs). High-dose corticosteroids remain the preferred initial therapy, given intravenously or orally, with plasma exchange considered in severe cases [7-10].

Oral prednisolone is typically continued for 24-36 months, as shorter courses increase the relapse risk. The tapering regimen starts at 50-60 mg for the first 2-4 months, reducing to 20-30 mg by 12 months, followed by a gradual taper. In elderly patients, glucocorticoid side effects require careful monitoring. Mycophenolate mofetil is often used as a steroid-sparing agent, but rapid tapering may trigger relapses. Cyclophosphamide has variable outcomes, whereas rituximab appears more effective, although long-term studies are needed [8-10].

The differential diagnosis of LGI1 encephalitis includes infectious encephalitis, such as herpes simplex virus (HSV) encephalitis, which often presents with seizures, fever, focal neurological deficits, and more extensive MRI changes compared with autoimmune encephalitis. Temporal lobe glioma can mimic mesial temporal swelling; however, autoimmune encephalitis typically has a gradual onset, and the swelling resolves with treatment. Creutzfeldt-Jakob disease (CJD) and other rapidly progressive dementias should be considered in chronic LGI1 antibody-positive patients; nevertheless, differences in clinical presentation, CSF analysis, and imaging usually facilitate differentiation. Postictal MRI changes may also resemble LGI1 encephalitis; therefore, careful clinical correlation is required [9,10].

In this article, we present a case of super-refractory status epilepticus caused by LGI1 encephalitis in a 61-year-old Saudi man who successfully responded to rituximab.

## Case presentation

We report a case of autoimmune limbic encephalitis (LE) associated with LGI1 antibodies in a 61-year-old Saudi retired man who presented with a three-week history of cognitive decline and recurrent right faciobrachial dystonic seizures, progressing to generalized tonic-clonic seizures and persistent hyponatremia. His other laboratory tests were within the normal range.

**Figure 1:**
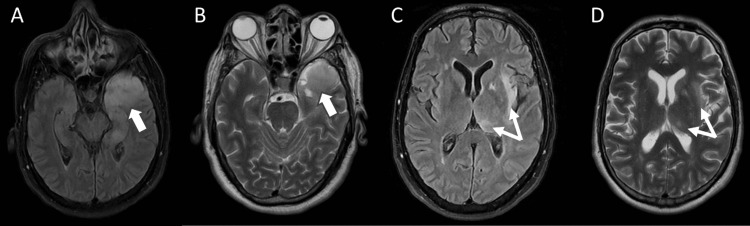
Magnetic resonance imaging (MRI) of the brain revealed extensive cortical and subcortical T2 and fluid-attenuated inversion recovery (FLAIR) hyperintensities in the left temporal lobe (A and B, thick arrows) and the left insular region, along with a focal hyperintense area in the left thalamus (C and D, thin arrows).

**Figure 2:**
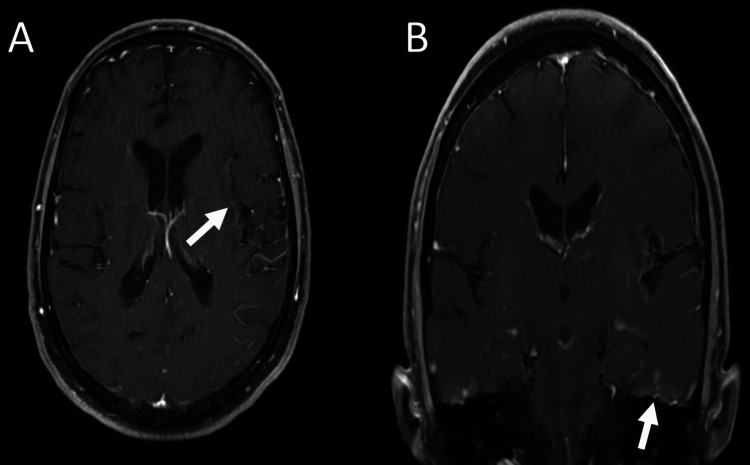
T1 post-gadolinium MRI of the brain. Axial and coronal sequences (A and B) revealed faint enhancement in the left temporal lobe and insular region, with associated leptomeningeal enhancement. MRI: magnetic resonance imaging

**Figure 3:**
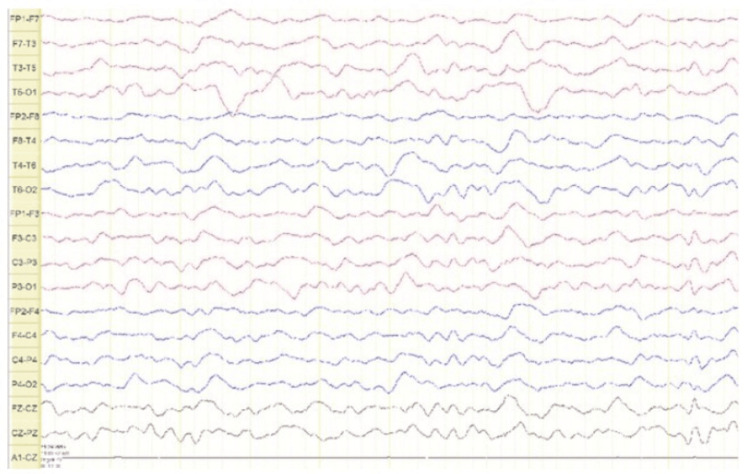
Electroencephalography showing diffuse slow background activity without electrographic seizures or epileptiform discharges.

The patient required intubation and sedation with midazolam, fentanyl, and propofol. Brain MRI (T2, fluid-attenuated inversion recovery {FLAIR}, and T1 post-gadolinium) revealed high signal intensity in the left temporal lobe and left insular region and faint enhancement (Figure 1 and Figure 2). Additionally, EEG revealed continuous symmetrical background slowing without electrographic seizures or epileptiform activity (Figure 3).

Cerebrospinal fluid (CSF) analysis revealed pleocytosis (28/μL; normal: <5/μL) with 100% mononuclear cells, an elevated protein level (97 mg/dL; normal: <45 mg/dL), and a normal glucose level. A multiplex polymerase chain reaction (PCR) panel, including testing for herpes simplex virus, was negative, whereas LGI1 antibodies were detected in both serum and CSF.

The clinical presentation, combined with the radiological findings and serum and CSF results, met the criteria for limbic encephalitis, primarily LGI1 encephalitis. Treatment was initiated with intravenous (IV) levetiracetam 1500 mg twice a day (BID), carbamazepine 400 mg twice a day, phenobarbital 100 mg twice a day, lacosamide 200 mg twice a day, and clonazepam 2 mg twice a day via a nasogastric tube, resulting in only partial seizure control. After excluding infections, the patient underwent five pulses of 1 g methylprednisolone and six sessions of plasma exchange (PE) but showed no significant improvement.

Due to persistent clinical seizures, the patient received three doses of intravenous rituximab (1000 mg IV), resulting in significant seizure control after the second cycle and a gradual clinical and functional recovery. He was subsequently enrolled in rehabilitation therapy, and no seizures were reported during the follow-up.

At six, 12, and 18 months, the patient remained ambulatory and seizure-free, which allowed for the gradual tapering of anti-seizure medications (ASMs) without any recurrence. He is currently maintained on lacosamide 100 mg BID and carbamazepine 400 mg BID.

The follow-up EEG, repeated in the outpatient setting, demonstrated normal background activity with no electrographic seizures or newly emerged epileptiform discharges and showed improvement in the previously observed diffuse slowing, correlating with the patient’s clinical recovery.

## Discussion

Limbic encephalitis (LE) is linked to a number of neuronal antibodies, including antineuronal nuclear antibody type 1 (anti-Hu); antineuronal nuclear autoantibody type 2 (ANNA-2), also known as anti-Ri; anti-Yo antineuronal antibody (anti-Yo); anti-Ma2; anti-amphiphysin; and anti-CV2/collapsin response mediator protein 5 (CRMP5). These antibodies are distributed throughout the nervous system and are linked to various neurological disorders that affect broader brain regions [11]. One of these is anti-LGI1 limbic encephalitis (anti-LGI1 LE), a unique type of autoimmune encephalitis that mainly affects the medial temporal lobe and causes seizures and memory loss. This disorder manifests as symptoms such as faciobrachial dystonic seizures (FBDSs), cognitive difficulties, and progressive deterioration over time [10,11]. Affected patients frequently exhibit hyponatremia, yet this remains a nonspecific finding [11]. In this case, the patient had typical symptoms such as low sodium levels, trouble sleeping, and abnormalities on MRI. There were also positive LGI1 antibodies in the blood and cerebrospinal fluid (CSF), which confirmed the diagnosis.

Autoimmune LE can be challenging to diagnose because it can show up in different ways. It may only affect the limbic system or spread to other parts of the brain, which makes the diagnosis uncertain [12]. Although insomnia has not been widely documented in anti-LGI1 LE, a study from France described a 65-year-old patient with this condition who presented with reversible sleep disturbances [11]. The relationship between LGI1 antibodies and sleep dysfunction remains unclear, and few studies have explored this association [13]. Neuropathological studies propose that an immune system targeting hippocampal neurons through cluster of differentiation 8+ (CD8+) T cells may cause this disease [14].

The early detection of FBDSs allows immunotherapy to begin immediately when it is most effective. Although there are no set rules for treatment, high-dose corticosteroids are usually used in the first place, along with intravenous immunoglobulin (IVIG), plasma exchange, or mycophenolate. However, if there is no clinical improvement within 1-2 weeks, second-line therapies such as plasmapheresis, cyclophosphamide, or rituximab should be considered [15]. Clinical status remains the most reliable indicator of treatment response, as antibody titers do not always correlate with disease progression or improvement. Moreover, it is still not clear how well steroid-sparing drugs such as mycophenolate and azathioprine work in the long term to stop diseases from returning [16].

Given the potential for severe disability, the early initiation of immunotherapy is recommended, even before confirmatory antibody results are available, as this approach may shorten disease duration and improve overall outcomes. However, to enhance treatment strategies, establish standardized therapeutic protocols, and better understand the mechanisms behind symptoms such as insomnia, further research is needed [12,16].

## Conclusions

LGI1 encephalitis is an uncommon autoimmune disorder that causes seizures, memory loss, and other neurological symptoms. The condition is triggered by antibodies targeting LGI1, a protein essential for nerve signal transmission between cells. Common symptoms include seizures, memory impairment, confusion, personality changes, and hyponatremia. Beyond these neurological manifestations, LGI1 encephalitis significantly impacts the quality of life, particularly due to cognitive dysfunction, which can lead to difficulties in attention, executive function, and problem-solving, affecting daily activities, social interactions, and occupational performance. Rituximab has shown effectiveness, especially in treatment-resistant cases, but larger studies are needed to confirm its efficacy, optimal dosing, and duration.
